# Surgical removal of the largest transgastric migrated gossypiboma: A case report

**DOI:** 10.1097/MD.0000000000039227

**Published:** 2024-08-23

**Authors:** Aya Haji Mohamad, Mohammad Al-Jawad, Abdulrazak Anadani, Hilal Matar, Ammar Niazi

**Affiliations:** a Faculty of Medicine, Aleppo University Hospital, University of Aleppo, Aleppo, Syria; b Faculty of Medicine, University of Aleppo, Aleppo, Syria; c Department of General Surgery, Faculty of Medicine, Aleppo University Hospital, Aleppo, Syria.

**Keywords:** case report, gastric migration, gossypiboma, surgical towel, textiloma

## Abstract

**Rationale:**

Gossypiboma is a term that refers to the condition of accidentally retained surgical gauze after surgeries. While many manifestations and complications are possible in this case, the migration of the retained gauze into the gastric cavity is one of the rarest. In this paper, we report the largest migrated surgical towel to the gastric cavity in the literature, measuring 90 cm × 90 cm.

**Patient concerns:**

A 33-year-old woman with recurrent epigastric pain unresponsive to treatment was referred to our hospital. She had undergone an open surgery cholecystectomy 11 years before admission during wartime in Syria.

**Diagnoses:**

Abdominal computed tomography with contrast showed a large mass in the stomach, indicating malignancy. However, upper gastrointestinal endoscopy revealed a gray–black foreign body occupying the entire gastric lumen, which indicated the presence of bezoar. Upon surgery, the final diagnosis of gastric gossypiboma was made; which was a retained surgical towel from the previous cholecystectomy that had fully migrated to the stomach and resembled both malignancy and bezoar upon investigation.

**Interventions:**

The patient underwent open surgery to excise the foreign body.

**Outcomes:**

The gossypiboma was successfully removed, and the patient was discharged 5 days after the operation without complications.

**Lessons:**

Retained surgical items, such as gossypiboma, can lead to significant medical complications. The migration of gossypiboma to the stomach, though rare, poses challenges in diagnosis and management, often requiring open surgical removal to prevent adverse outcomes. Early detection and intervention are crucial to avoiding associated morbidity and mortality. It is important to consider gossypiboma in patients with unexplained abdominal pain following surgery and to emphasize meticulous sponge counting to prevent this complication.

## 1. Introduction

Retained surgical item is a rare yet an important complication that may occur in any type of open surgical procedure, causing potential harm to the patient and medicolegal consequences for surgeons and hospitals. The most commonly retained surgical items are surgical sponges or gauzes due to their frequent use and the difficulty in distinguishing them from the surrounding tissue after they are soaked in blood.^[1,2]^ The medical condition of retained surgical gauze is known as “gossypiboma” which is derived from the Latin word “Gossypium” meaning cotton, and the Swahili word “boma” meaning place of concealment. Other terms to describe the situation are “textiloma,” “gauzoma,” and “muslinoma.”^[3]^ The estimated incidence of retained medical items in abdominal cavity surgeries is as high as 1 in 1000 to 1 in 1500.^[4]^

A very rare but serious complication of gossypiboma is internal transmural migration into the gastrointestinal tract that may lead to intestinal or gastric outlet obstruction, malabsorption, or gastrointestinal bleeding.^[5]^

It is unlikely for a large gauze or surgical towel to be retained and moreover to migrate into the gastrointestinal tract. In this paper, we report a case of a 33-year-old female with the largest reported gossypiboma filling the gastric lumen and mimicking a bezoar, diagnosed after 11 years.

## 2. Case presentation

A 33-year-old woman with a body mass index (BMI) of 30.4 presented to our hospital with recurrent epigastric pain unresponsive to treatment, nausea without vomiting, and a retrosternal burning sensation. She had a surgical medical history of appendectomy in 2003, ovarian laparocystectomy in 2008, and open surgery cholecystectomy in 2012 during wartime in Syria. After the open surgery cholecystectomy, she suffered from an abscess in the scar site for 6 months. The abscess was drained and treated with antibiotics and honey dressings. She lost weight for 3 years without abdominal distention; after that, she gained weight for 4 years until she had weight stability.

**Figure 1.**
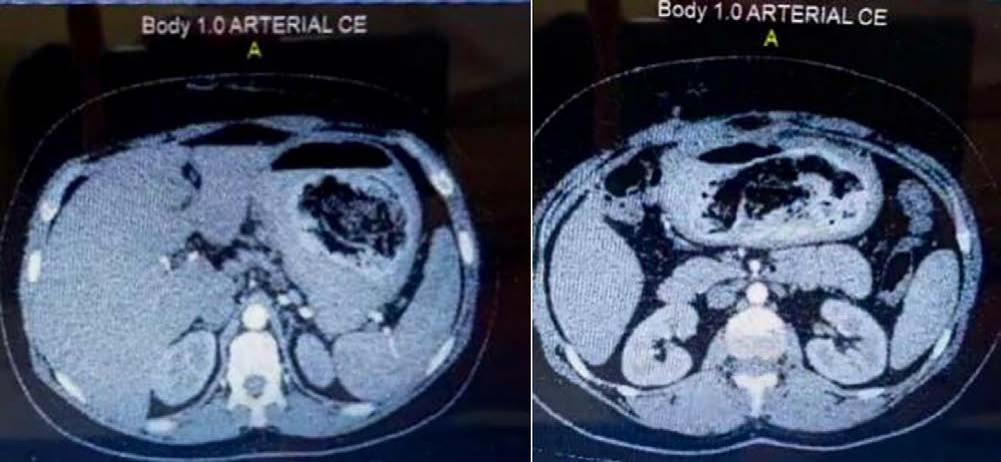
CT with contrast showcasing a large unattached heterogenous mass in the gastric lumen, with reactive diffuse and irregular thickening of the gastric mucosa, indicating a foreign body or malignancy. CT = computed tomography.

**Figure 2.**
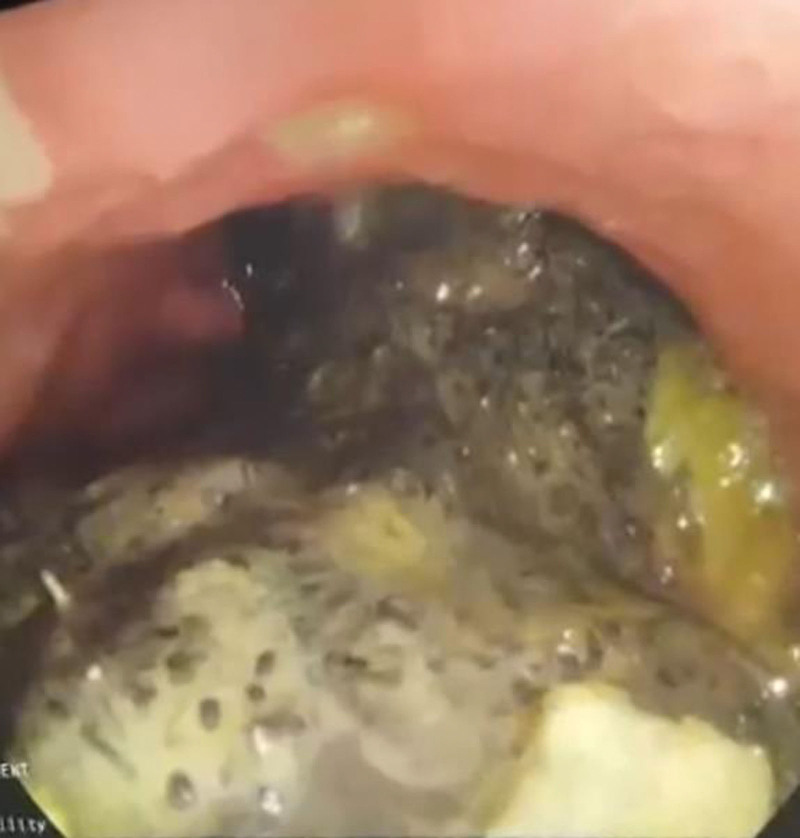
A gray-black mass compatible with bezoar was seen in the stomach via upper gastroendoscopy.

**Figure 3.**
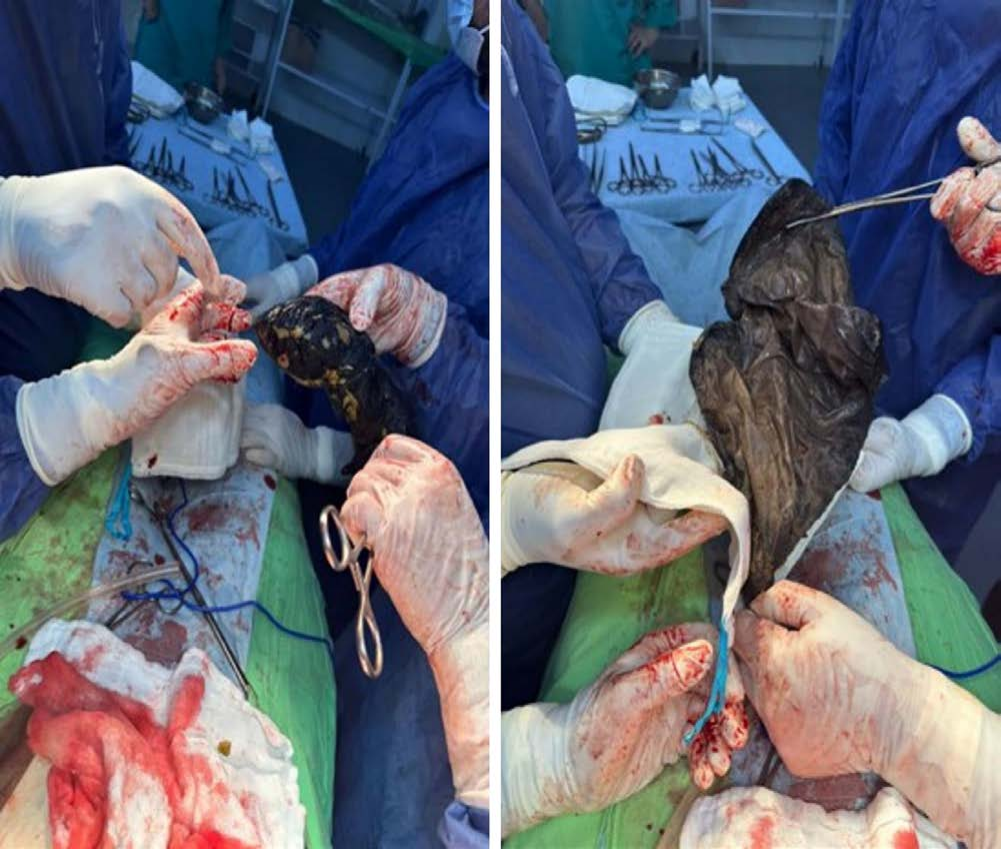
Surgical removal of gossypiboma from the stomach.

**Figure 4.**
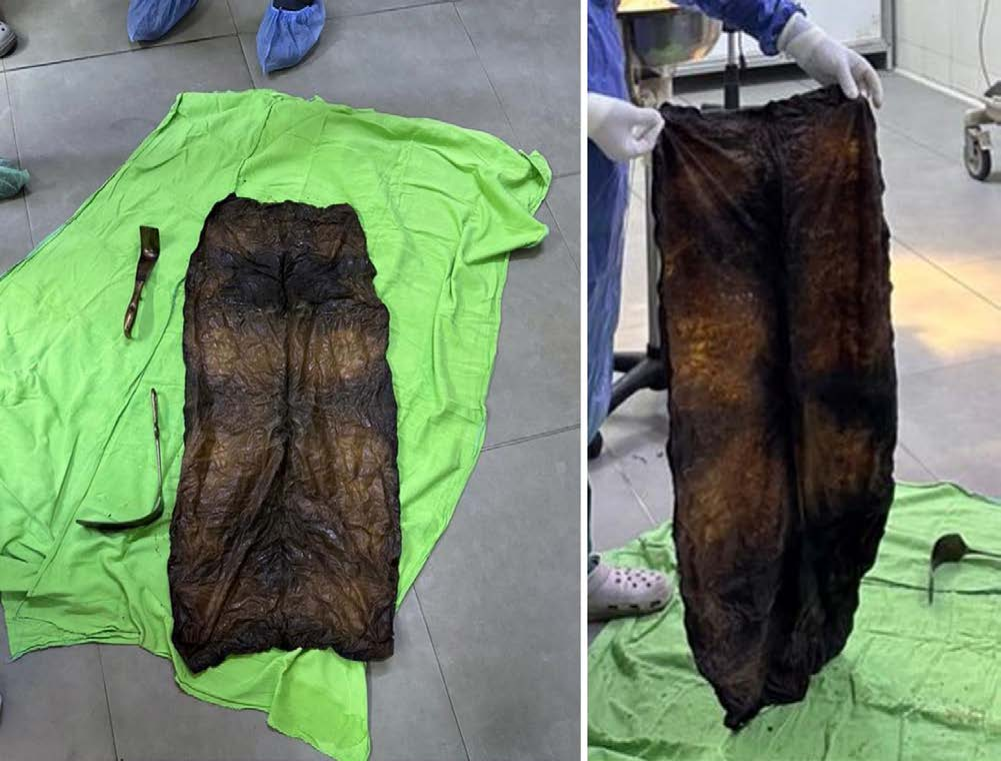
View of the retained surgical towel after surgical removal, 90 cm × 90 cm long in size. The towel was folded and sewn from the edges.

Plain abdominal radiography and ultrasound were unremarkable. Abdominal computed tomography (CT) with contrast revealed a large, unattached heterogenous mass in the stomach, indicating a foreign body or malignancy (Fig. 1). Upper gastrointestinal endoscopy showed a gray-black foreign body occupying the entire stomach, and a diagnosis of bezoar was made. Large gastric ulcers FOREST III classification were seen in the pyloric antrum and smaller ulcers in the body. Multiple ulcers FOREST III classification were also seen in the duodenal bulb (Fig. 2). The mass could not be removed through endoscopy due to its large size; therefore, the patient was referred to the general surgery department for open surgery. A complete blood count (CBC) showed slightly decreased hemoglobin levels of 10.2 g/dL and normal white blood cells, international normalized ratio (INR), and platelets levels. The chemistry panel showed decreased Na levels of 131 mEq/L. The patient underwent open surgery to remove the large foreign body. After Adhesiolysis, a perforation was found in the stomach body covered with omentum. Gastrotomy was performed at the perforation site, followed by the removal of the foreign body. We realized that the foreign body was a gossypiboma misdiagnosed as bezoar (Fig. 3). The perforation was closed. The gossypiboma was a large retained surgical towel from a previous open cholecystectomy that migrated completely to the stomach, with a size of 90 cm × 90 cm (Fig. 4). The entry point of the gossypiboma was sealed by the omentum; therefore, there was no peritonitis. The postoperative period elapsed without complications, and the patient was discharged from the hospital 5 days after the surgery.

## 3. Discussion

Discovering a retained surgical item after an intra-abdominal surgery can lead to significant medical and legal complications. Although the reported occurrence of this issue ranges from 1 in 1000 to 1 in 1500 cases of intra-abdominal surgeries, it is believed to be more frequent than what is officially documented.^[6]^

Among forgotten surgical objects, surgical sponges are the most frequently encountered, with the abdomen being the most affected anatomical cavity, representing 56% of cases. The thorax and pelvis represent about 11% and 8% of cases, respectively.^[7]^

Gossypiboma elicits 2 types of foreign body reactions. The first is usually asymptomatic and is characterized by an aseptic fibrinous response, which contributes to the formation of adhesions and encapsulation around the gossypiboma. As a result, it can be calcified, disrupted, partially absorbed, or diffused. The second type involves an exudative reaction, leading to an inflammatory response which can be symptomatic. The inflammation surrounds the foreign body and leads to the development of an abscess and the erosion of surrounding tissues. This results in foreign body sinus or fistula formation, or migration of the foreign body into the gut.^[8]^

The occurrence of migration is infrequent in comparison to abscess formation. This process undergoes 4 stages: foreign body reaction, secondary infection, mass formation, and remodeling. Upon complete migration, the migration site closes. The small intestine is particularly susceptible to transmural migration due to its thin wall and large outer surface area. In contrast, the stomach, with its thicker wall and small outer surface, is an atypical site for migration.^[9]^

To the best of our knowledge, only 9 cases of gastric migration were reported in the literature; 8 of them were associated with a previous hepatobiliary operation.^[8]^ In our case, the patient had an emergency open cholecystectomy 10 years before the diagnosis of the gossypiboma.

The clinical presentation is variable, depending on the anatomical site where the gauze is retained and the type of reaction it provoked. It may remain asymptomatic. However, if the gauze migrates into the lumen of the stomach, small intestine, or colon, patients may have symptoms such as nausea, vomiting, and obstruction.^[10,11]^ It can be acute after granuloma and abscess formation, or chronic accompanied by adhesions.^[12]^ Our patient initially suffered from an abscess in the acute phase; afterwards, she had chronic symptoms due to gastric migration of the gossypiboma, Which, despite its large size, did not lead to gastric obstruction, and as a result, it was not diagnosed until 11 years later.

The most prominent risk factors include the performance of complex and emergency surgical procedures, open abdominal procedures, higher BMI, involvement of multiple surgical teams, and utilization of a large number of surgical instruments.^[11]^

It is also important to highlight that war influences patient care and may lead to medical errors during surgeries. Custović et al^[13]^ reported 14 gossypiboma cases, of which 9 cases were during wartime in Croatia; pointing out the association between war and an increased incidence of gossypiboma. In our case, the causative operation of the gossypiboma was an emergency open cholecystectomy, which took place in 2012 during wartime in Syria, and was accompanied by a shortage of medical equipment and a constant state of stress among the surgical team.

Imaging studies are helpful in diagnosis. Plain X-ray exhibits radio-opaque material within the body. However, it lacks both sensitivity and specificity in accurately diagnosing gossypiboma, especially if the material twists, folds, or disintegrates over time. Consequently, CT is considered the investigation of choice. CT demonstrates high sensitivity and reasonable specificity in the diagnosis. The most characteristic finding of gossypiboma on CT is a well-defined mass with a dense central part and a hyperdense, enhancing capsule wall.^[8,9]^

However, these radiographic findings are inconclusive, especially in chronic cases when the mass does not have radiological marker. Thus, it can be misdiagnosed as tumor or bezoar, as seen in our case.^[14]^

In the last decade, endoscopy has been considered a diagnostic and therapeutic tool. Obeidat et al^[8]^ indicate that it was diagnostic in 73% of a total of 30 cases of gossypiboma. In our case, the patient underwent an upper endoscopy because there was a suspicion of a malignant tumor. On endoscopy, a huge gray-black mass with an irregular surface was seen occupying the entire stomach, demonstrating the findings of bezoar. The mass could not be removed through endoscopy due to its size; therefore, the patient was referred to the surgical department.

While prevention is the most effective approach, open surgery is considered the best therapy for a retained gossypiboma in the abdomen. Delay in treatment can result in a mortality rate of 10%.^[8]^

Although few successful cases of upper gastrointestinal removal of the gossypiboma were reported^[10,11]^; in our case, the mass was occupying the entire stomach and was initially diagnosed as bezoar. Therefore, open surgical removal was the best option, and the final diagnosis of 90 cm × 90 cm gossypiboma was made.

To the best of our knowledge, this is the largest size of migrating surgical towel to the stomach documented in the medical literature.

## 4. Conclusion

A differential diagnosis of gossypiboma should be considered in any patient with a surgical history who is complaining of abdominal pain and is unresponsive to treatment. This condition can be avoided with proper pre and postoperative counting of sponges and following preventive measure

## Author contributions

**Writing – original draft:** Aya Haji Mohamad, Mohammad Al-Jawad, Abdulrazak Anadani.

**Writing – review & editing:** Aya Haji Mohamad, Hilal Matar.

**Supervision:** Ammar Niazi.
